# Quorum Sensing Regulates the Production of Methanethiol in *Vibrio harveyi*

**DOI:** 10.3390/microorganisms12010035

**Published:** 2023-12-24

**Authors:** Tiantian Zhou, Jinyan Wang, Jonathan D. Todd, Xiao-Hua Zhang, Yunhui Zhang

**Affiliations:** 1 College of Marine Life Sciences, Ocean University of China, 5 Yushan Road, Qingdao 266003, China; oucttzhou@163.com (T.Z.); wangjylc@163.com (J.W.); xhzhang@ouc.edu.cn (X.-H.Z.); 2 School of Biological Sciences, University of East Anglia, Norwich Research Park, Norwich NR4 7TJ, UK; jonathan.todd@uea.ac.uk; 3 Institute of Evolution & Marine Biodiversity, Ocean University of China, 5 Yushan Road, Qingdao 266003, China; 4 Laboratory for Marine Ecology and Environmental Science, Laoshan Laboratory, Qingdao 266071, China

**Keywords:** MeSH, DMS, quorum sensing, *Vibrio harveyi*, MegL

## Abstract

Methanethiol (MeSH) and dimethyl sulfide (DMS) are important volatile organic sulfur compounds involved in atmospheric chemistry and climate regulation. However, little is known about the metabolism of these compounds in the ubiquitous marine vibrios. Here, we investigated MeSH/DMS production and whether these processes were regulated by quorum-sensing (QS) systems in *Vibrio harveyi* BB120. *V. harveyi* BB120 exhibited strong MeSH production from methionine (Met) (465 nmol mg total protein^−1^) and weak DMS production from dimethylsulfoniopropionate (DMSP) cleavage. The homologs of MegL responsible for MeSH production from *L*-Met widely existed in vibrio genomes. Using BB120 and its nine QS mutants, we found that the MeSH production was regulated by HAI-1, AI-2 and CAI-1 QS pathways, as well as the *luxO* gene located in the center of this QS cascade. The regulation role of HAI-1 and AI-2 QS systems in MeSH production was further confirmed by applying quorum-quenching enzyme MomL and exogenous autoinducer AI-2. By contrast, the DMS production from DMSP cleavage showed no significant difference between BB120 and its QS mutants. Such QS-regulated MeSH production may help to remove excess Met that can be harmful for vibrio growth. These results emphasize the importance of QS systems and the MeSH production process in vibrios.

## 1. Introduction

The volatile organosulfur compounds (VOSCs) methanethiol (MeSH) and dimethyl sulfide (DMS) are important intermediates of the global sulfur cycle that impact atmospheric chemistry and climate [1,2]. Known as a signaling molecule [3], DMS is present in the Earth’s surface oceans at levels equivalent to ~15–47 Tg S and is emitted into the atmosphere at levels of ~28.1 (17.6–34.4) Tg S [4]. MeSH, a major sulfur source for sulfoproteins in marine bacteria [5], is also ubiquitous in marine systems (0.02–3.49 nM levels) and is emitted into the atmosphere at ~17% of the levels of DMS [6,7,8]. Hydrogen sulfide (H_2_S) and MeSH can be *S*-methylated to yield DMS, via the action of enzymes such as MddA (methanethiol *S*-methyltransferase, EC 2.1.1.334), by diverse bacteria, haloarchaea and algae [9,10]. Atmospheric MeSH and DMS oxidation products can act as cloud condensation nuclei (CCN) and influence radiative properties and climate [11]. Additionally, DMS is a chemosignal that can attract diverse organisms including bacteria, (in)vertebrates and mammals [12,13,14].

In marine systems, DMS and MeSH are produced from microbial catabolism of the abundant osmolyte dimethylsulfoniopropionate (DMSP) via DMSP lysis and demethylation pathways [15], respectively. The genetic potential for this DMSP catabolism is prevalent in marine systems, with >20% of marine bacteria containing the demethylation (*dmd*) or cleavage (*ddd*) genes [16]. MeSH can also be generated from the demethylation of methionine (Met), catalyzed by *L*-Met gamma-lyase (MegL) (EC 4.4.1.11) [17], which is widely distributed in diverse bacteria including *Pseudomonas* sp. [17,18], *Brevibacterium linens* [19], *Citrobacter freundii* [20] and *Streptomyces avermitilis* [21]. DMS itself can be demethylated to form MeSH, which is mediated by DMS monooxygenase DmoA (EC 1.14.13.131) in *Hyphomicrobium sulfonivorans* [22]. Notably, MeSH is a key source of reduced sulfur assimilated by marine bacteria, including SAR11 clade bacteria [23]. Thus, marine bacteria play vital roles in the production and cycling of DMS and MeSH, but the regulation of these processes remains largely unknown.

Quorum sensing (QS) is a widespread cell-to-cell communication mechanism that enables bacteria to control gene expression in response to cell density [24,25,26]. It relies on the production, release, accumulation and detection of signaling autoinducers [27,28,29]. Acylated homoserine lactone (AHL) and Autoinducer-2 (AI-2) are the most widely studied autoinducers [30,31]. QS controls carbon, nitrogen, sulfur and primary metabolism in bacteria [32,33]. For instance, specific *Acidithiobacillus ferrooxidans* AHLs can promote their attachment to elemental sulfur and upregulate genes involved in sulfur oxidation [34,35]. However, whether QS also plays a role in bacterial organic sulfur metabolism is not clear.

**Figure 1:**
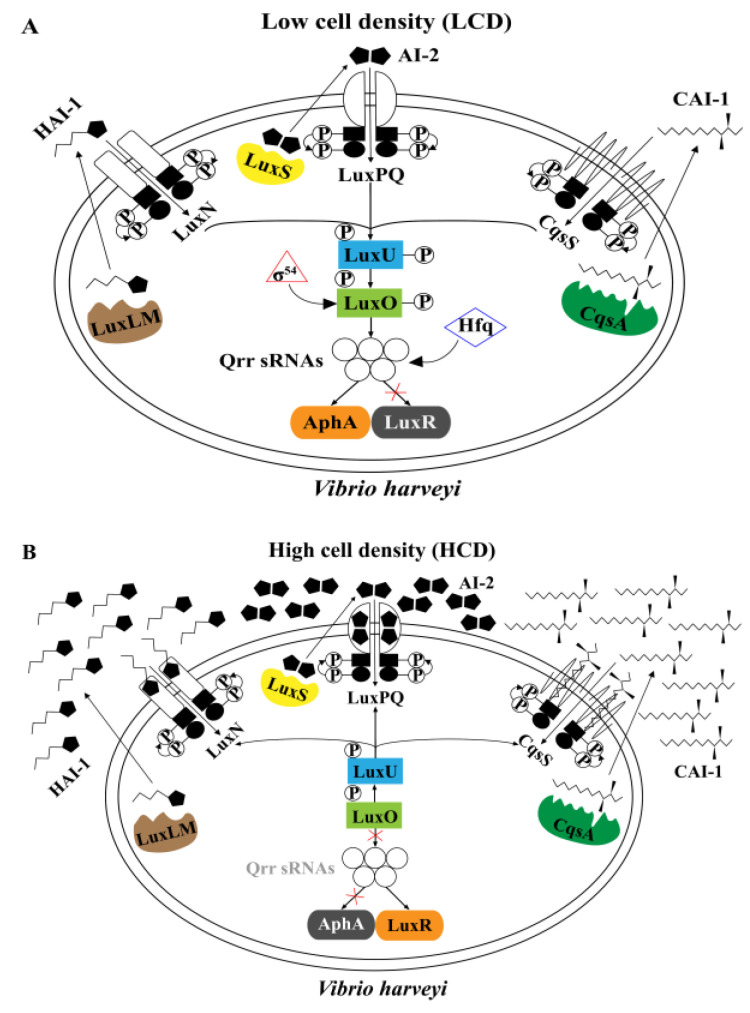
Model of the *Vibrio harveyi* quorum-sensing (QS) systems. (**A**) QS systems in *V. harveyi* at low cell density (LCD). (**B**) QS systems in *V. harveyi* at high cell density (HCD). *V. harveyi* has three parallel QS systems that regulate the expression of LuxR and AphA under different conditions. LuxLM, LuxS and CqsA enzymes synthesize the signal molecules HAI-1, AI-2 and CAI-1, respectively. These autoinducers are detected by LuxN, LuxQ and CqsS bi-component receptor proteins, respectively. Detection of AI-2 by LuxQ requires the periplasmic protein LuxP. The kinase activity dominates when *V. harveyi* is at LCD, while the receptors transform into phosphatases when at HCD. At LCD, the three receptors autophosphorylate and transfer phosphate to LuxO via LuxU. Under the direction of σ^54^ factor, phosphorylated LuxO activates the production of the quorum-regulated small RNAs (Qrr sRNAs). Together with the chaperone Hfq, Qrr sRNAs repress translation of LuxR, and activate translation of AphA that regulates the individual behaviors including synthesis of flagella and pilus, which further influence the motility of bacteria [40,41]. At HCD, the flow of phosphate reverses and LuxO dephosphorylates, inhibiting the production of Qrr sRNAs and AphA and stimulating the translation of LuxR.

The well-studied QS systems in the model organism *Vibrio harveyi* consist of three parallel QS systems, LuxLM/N, LuxS/PQ and CqsA/S [36] (Figure 1), which produce and respond to their cognate autoinducers (HAI-1, AI-2 and CAI-1, respectively) [37,38]. HAI-1 is an AHL-type molecule and acts as the primary autoinducer in *V. harveyi*. The LuxO protein is central in this signal transduction cascade; phosphorylated LuxO can inhibit the production of the transcriptional regulator LuxR indirectly. The quorum-regulated small RNAs (Qrr sRNAs), σ^54^ factor and the chaperone Hfq are also involved in the regulation of LuxR. LuxR can further induce bioluminescence and the production of virulence factors [36] and repress the type III secretory system [39].

*Vibrio* spp. are ubiquitous marine bacteria with high metabolism flexibility, but their potential role in organic sulfur metabolism is seldom discussed. In the current study, we show that marine vibrios were able to produce MeSH and DMS and investigate the genetic basis for the production of these climate-active gases. Furthermore, we investigate whether MeSH and DMS production is regulated by QS systems in *V. harveyi* BB120 [42] and the potential molecular basis for this.

## 2. Materials and Methods

### 2.1. Strains and Cultivation Conditions

The detailed descriptions of *V. harveyi* BB120 and its QS mutants are summarized in Table S1. All the strains used in the study were kindly donated by Professor Bonnie Lynn Bassler (Princeton University). These strains were routinely cultured in Marine Basal Medium (MBM) [43] or Marine Agar (MA, BD Difco, Sparks, MD, USA) with the supplement of corresponding antibiotics for the mutants.

### 2.2. Quantification of MeSH and DMS Production

Strains were cultivated in MBM minimal medium (35 PSU) supplemented with a mixed carbon source (10 mM from a 1 M stock of 200 mM succinate, glucose, pyruvate, sucrose and glycerol). A 1 mM amount of *L*-Met or 1 mM DMSP, respectively, was supplemented as the substrate for MeSH/DMS. After 24 h, 200 μL of cultures was added to 2 mL vials. Those vials were crimped immediately and incubated at 28 °C overnight in the dark. The headspace MeSH and DMS produced were quantified by gas chromatography (GC) using a flame photometric detector (Agilent 7890B GC fitted with a 7693A autosampler, Santa Clara, CA, USA) and an HP-INNOWax 30 m × 0.320 mm capillary column (Agilent Technologies J&W Scientific, Santa Clara, CA, USA). DMS calibration curves were produced as described by Curson et al. [44]. The emissions of MeSH and DMS minus those detected in the control groups were considered to be MeSH and DMS production, respectively. To determine the cell protein content, a Thermo Multiskan Go microplate reader (Waltham, MA, USA) was used for the measurement of the absorbance in the Bradford assays. MeSH and DMS production were expressed as nmol per mg total protein. Three biological replicates were used for each sample, and the results shown are means of these replicates. Changes of MeSH- and DMS-releasing activities of the strains were compared by Student’s *t*-test.

### 2.3. Identification of Potential Proteins Related to MeSH and DMS Metabolism

To explore the potential enzymes involved in the MeSH and DMS production in *V. harveyi* BB120, ratified proteins related to *L*-Met metabolism and the DMSP cycling were used as training protein sequences for BLASTp according to the references [21,45,46,47,48,49,50]. The genome of *V. harveyi* BB120 was searched against the above ratified proteins, and the potential functional proteins related to MeSH and DMS metabolism were recognized with specific standards as described previously [51,52,53]. The conserved domains of MegL in *V. harveyi* BB120 were characterized by NCBI conserved domain search.

Additionally, the complete genomes of other representative *Vibrio* species were obtained from NCBI for the identification of the potential functional proteins involved in *L*-Met and DMSP cycling as described above. *e*-value cut-offs were based on levels of identity shared by functionally verified protein sequences and are listed in Table S2. For those proteins with no reference, an *e*-value cut-off < *e*^−5^ was set as the standard for the preliminary screening. To further confirm whether the selected proteins were clustered with the characterized, phylogenetic trees based on their sequences were built, and the related information of proteins clustered or with higher identity and coverage and lower *e*-value is also listed in the table. Furthermore, those proteins with higher identity were selected for the later construction of phylogenetic trees. Maximum-likelihood phylogenetic trees (Bootstrap = 1000) based on protein sequences were constructed using MEGA X [54] with a Jones–Taylor–Thornton (JTT) model, and the visualization of trees was realized by Interactive Tree Of Life (iTOL) v5 [55].

### 2.4. Growth of V. harveyi and Its Mutants with L-Met

*V. harveyi* wild-type (WT) BB120, *luxS*^−^ mutant MM30 and *luxLM*^−^/*luxS*^−^ mutant MM77 were cultured as described above. After incubation at 28 °C overnight, cultures were transferred to MBM and cultivated with conditions of 170 rpm under the same circumstances as described above. Then, OD_600_ was adjusted to 0.3 the next day, and cultures were inoculated with the ratio of 10% to a 96-well plate, to which 180 µL fresh MBM was added in each well. The growth curves of the strains with *L*-Met added were depicted by the continuous measurement at 28 °C for 24 h of a Thermo Multiskan Go microplate reader in the interval of 1h to evaluate the impact of *L*-Met on their growth. Three biological replicates were used for each strain, and the results shown are means of these replicates.

### 2.5. MeSH Production with Autoinducer and Quorum-Quenching Enzyme

To test the influence of AI-2 on MeSH production of *V. harveyi*, WT BB120, *luxS*^−^ mutant MM30 and *luxLM*^−^/*luxS*^−^ mutant MM77 were routinely cultured in MBM supplemented with *L*-Met for 24 h. Then, the bacterial suspension was transferred to the vials that contained fresh MBM with AI-2 added at a final concentration of 1 μM. Detection was conducted after incubation overnight as described above.

To investigate the influence of HAI-1 on MeSH production of *V. harveyi* BB120, quorum-quenching (QQ) enzyme MomL, which can degrade varied kinds of AHLs, was used. The MomL protein of *Muricauda olearia* was expressed in a heterologous manner in *Escherichia coli* and purified as described by Tang et al. [56]. Strain BB120 was routinely cultured as described above in the MBM with 1.5 mg/mL or 15 mg/mL purified MomL, and the MeSH production was quantified after 24 h. Three biological replicates were used for each sample, and the results shown are means of these replicates.

## 3. Results

### 3.1. MeSH and DMS Production by Vibrio harveyi BB120

**Figure 2:**
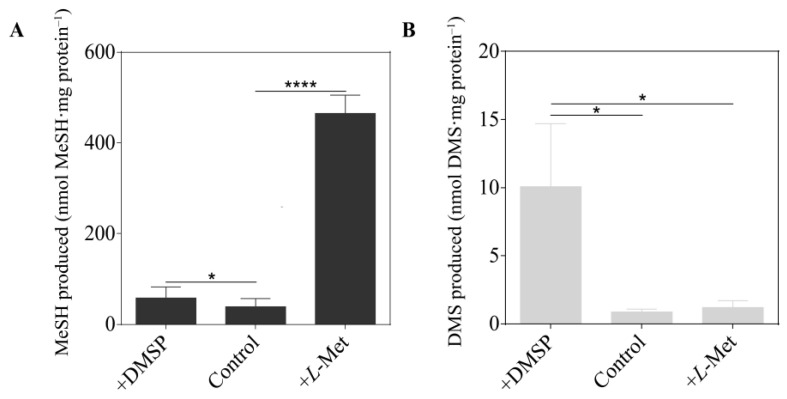
Production of sulfur gas by *Vibrio harveyi* BB120 cultured in MBM under different treatments. (**A**) Production of MeSH by *V. harveyi* BB120. (**B**) Production of DMS by *V. harveyi* BB120. The data are shown as the mean ± standard deviation (SD). The differences between the experimental groups and the control groups were calculated by Student’s *t*-test. *, *p* < 0.05 in Student’s *t*-test. ****, *p* < 0.0001 in Student’s *t*-test.

*V. harveyi* BB120 produced 38 nmol MeSH mg total protein^−1^ when cultured in MBM with no methylated sulfur compounds added (Figure 2). This MeSH production was >10-fold increased by addition of *L*-Met (465 nmol MeSH mg total protein^−1^) but not by that of DMSP, implying that *V. harveyi* lacked a DMSP demethylation pathway and contained MegL. Notably, addition of *L*-Met did not enhance DMS production by *V. harveyi*, implying that this model vibrio lacked a MeSH-dependent DMS production pathway. However, *V. harveyi* did display weak DMSP cleavage activity, producing 10 nmol DMS mg total protein^−1^ from DMSP, a phenotype which has never been reported in any vibrios. Similar trends were observed when the above experiments were repeated in nutrient-rich media (MA) (Figure S1).

### 3.2. Candidate Proteins Involved in MeSH and DMS Cycling in Vibrio

VH2527 (WP_005432739.1), which is annotated as *O*-succinylhomoserine (thiol)-lyase, was found to be the potential MegL in *V. harveyi* BB120, sharing higher similarity with the MegLs characterized (identity > 40%, *e*-value < *e*^−80^, coverage > 90%) (Table S3). The common conserved domain predicted in these MegLs belongs to the AAT_I superfamily. Within the domain, several functional elements were recognized, including the catalytic residue and pyridoxal 5′-phosphate binding site, which are two typical structural characteristics of MegLs [57]. Additionally, a substrate–cofactor binding pocket and homodimer interface were also found in them. All the functional elements mentioned above allow MegL activities. For DMSP catabolism, *V. harveyi* BB120 was found to lack a *dmdA* gene that encodes the primary enzyme of the bacterial DMSP demethylation pathway, which is consistent with the data above. Except for MegL, two potential DorAs, which served as dimethylsulfoxide (DMSO) reductase (EC 1.8.5.3) or trimethylamine *N*-oxide (TMAO) reductase (EC 1.7.2.3) and were in charge of the transformation from DMSO to DMS, VH851 (TMAO reductase, WP_012127641.1) and VH4908 (molybdopterin-dependent oxidoreductase, WP_012129114.1) (identity > 40%, *e*-value = 0.0, coverage > 98%), were also found in *V. harveyi* BB120 (Table S3).

**Figure 3:**
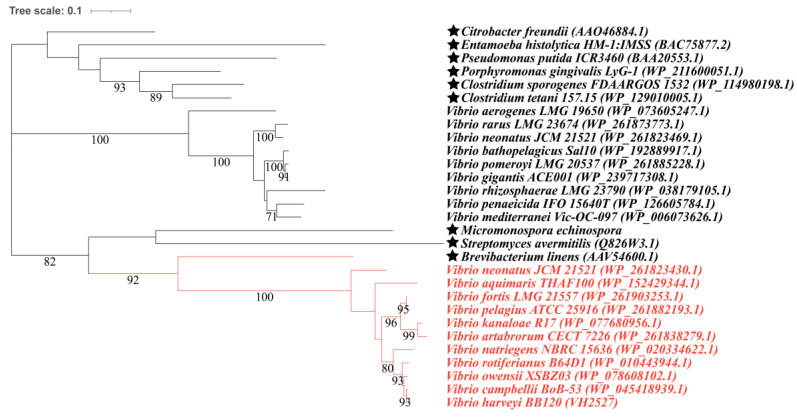
Maximum-likelihood phylogenetic tree of MegLs in *Vibrio*. Ratified MegLs alongside the proteins of the representative vibrios available from NCBI that shared higher identity (top 10) with them were used for phylogenetic tree construction. Proteins experimentally confirmed to produce MeSH are marked with a black star, and those in red are potential MegLs. Bootstrap support for nodes is marked.

To further investigate the MeSH and DMS metabolism in vibrios, 51 complete genomes of different species in the *Vibrio* genus were selected to study the distribution of proteins related to MeSH and DMS metabolism (Table S4). All of the strains above contained MegL, which indicates that MeSH production from *L*-Met is universal in vibrios. As the maximum-likelihood (ML) tree shows, MegLs in vibrios clustered with that of *Brevibacterium linens*, which suggests a closer relationship between them (Figure 3). Despite lacking the core enzyme of DMSP demethylation (DmdA, annotated as dimethylsulfoniopropionate demethylase, EC 2.1.1.269), five vibrios, which accounted for approximately 10% of those studied, were predicted to contain enzymes (DmdB and AcuH, annotated as 3-methylmercaptopropionyl-CoA ligase, EC 6.2.1.44 and enoyl-CoA hydratase) enabling the catabolism of MMPA (3-methylmercaptopropionate), which is the primary catabolite of DMSP demethylation [58] (Figure S2). With regard to the known DMSP lyases (EC 4.4.1.3), there was no potential DddL, DddQ, DddW, DddY, DddK or DddX [59] found in the selected strains (Table S4). However, *V. ostreae* OG9-811 was found to comprise DddD, which clustered with that of *Marinomonas* sp. MWYL1 (WP_012071702.1), and the similarity between them reached 76.562% (*e*-value = 0.0); DddPs in the other three vibrio genomes were in the same branch with that of *Oceanimonas doudoroffii* ATCC 27,123 (EC 4.4.1.3), sharing a similarity of over 79% (*e*-value = 0.0, coverage > 99%) (Figure S3). Whether these Ddd proteins are functional or not is yet to be determined. In terms of the conversion between DMS and DMSO, no Tmm (flavin-containing monooxygenase, EC 1.14.13.8) was found, and DdhA (4Fe-4S dicluster domain-containing protein, WP_060833690.1) was only found in two vibrio genomes (Figure S4), while DorA was distributed widely in up to ~75% of the selected genomes (Figure S5 and Table S4). Vibrios lacked the pathway allowing transformations between MeSH and DMS since no MddA or DmoA was found in their genomes. That they had no MTO-like protein (methanethiol oxidase, EC 1.8.3.4) indicated that vibrios are unable to transform MeSH into HCHO (Table S4).

### 3.3. QS Mutants of V. harveyi BB120 Differ in MeSH Production

**Figure 4:**
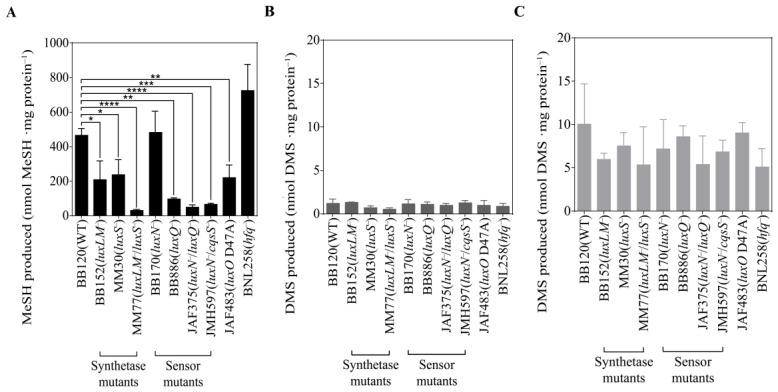
Production of sulfur gas by *Vibrio harveyi* BB120 and its 9 mutants cultured in MBM with *L*-Met/DMSP added. (**A**) Production of MeSH by *V. harveyi* BB120 and its 9 mutants cultured in MBM with *L*-Met added. (**B**) Production of DMS by *V. harveyi* BB120 and its 9 mutants cultured in MBM with *L*-Met added. (**C**) Production of DMS by *V. harveyi* BB120 and its 9 mutants cultured in MBM with DMSP added. The data are shown as the mean ± standard deviation (SD). The differences between the experimental groups and the control groups were calculated by Student’s *t*-test. *, *p* < 0.05 in Student’s *t*-test. **, *p* < 0.01 in Student’s *t*-test. ***, *p* < 0.001 in Student’s *t*-test. ****, *p* < 0.0001 in Student’s *t*-test.

Having established that *V. harveyi* BB120 produces MeSH and DMS via MegL and unidentified DMSP lyase(s), we investigated whether these processes were regulated by QS. The MeSH and DMS production phenotypes of wild-type *V. harveyi* BB120 and gene knockout mutants in their varied QS pathways were quantified (Figure 4). Compared with the wild type (WT), the production of MeSH produced by the mutants BB152 [60] and MM30 [61], in which *luxLM* and *luxS* were knocked out, fell by ~50% to 208 and 237 nmol MeSH mg total protein^−1^, respectively (Figure 4A). Furthermore, MeSH production by strain MM77 [62], mutated in both *luxLM* and *luxS*, dramatically reduced to 6% of the WT level (30 nmol MeSH mg total protein^−1^). These results indicated that both HAI-1 and AI-2 autoinducers may be involved in the regulation of MeSH production of *V. harveyi*.

Mutant BB170 [63], in which the sensor protein LuxN is non-functional, showed no significant difference from the WT in terms of MeSH production, which implied that *luxN* was not responsible for the modulating of MeSH production. In contrast, a mutation in the sensor *luxQ* gene (strain BB886, [60]) caused a ~80% reduction in its *L*-Met-dependent MeSH production (95 nmol MeSH mg total protein^−1^) compared to WT. When *luxN* and *luxQ* were both mutated in JAF375 [64], MeSH was reduced even further to ~10% of the WT (48 nmol MeSH mg total protein^−1^). The CqsS protein was also likely to be involved in the regulatory cascade controlling *L*-Met-dependent MeSH production since the *luxN*/*cqsS* double mutant strain JMH597 [37] showed a significant ~86% loss of this activity compared to the WT (64 nmol MeSH mg total protein^−1^). A strain mutated in *luxO* (JAF483, [64]), which acts as the central regulator of the QS cascade, also showed ~50% reduced *L*-Met-dependent MeSH production (221 nmol MeSH mg total protein^−1^) when compared with WT. Finally, mutated in the RNA chaperone Hfq, which can destabilize the downstream regulator *luxR* [65], BNL258 produced ~1.6-fold more MeSH from *L*-Met than WT (723 nmol MeSH mg total protein^−1^). These results indicated that the complete QS cascade participated in regulating MeSH production of *V. harveyi* BB120.

However, there was no significant impact of any QS gene knockout on the levels of DMS produced from the addition of *L*-Met (Figure 4B), which might be attributed to the lack of a MeSH-dependent DMS production pathway. Additionally, DMS produced through the DMSP lysis pathway by *V. harveyi* BB120 and its QS mutants showed no significant changes (Figure 4C).

### 3.4. Signal Molecules Affect MeSH Production in Vibrio harveyi

**Figure 5:**
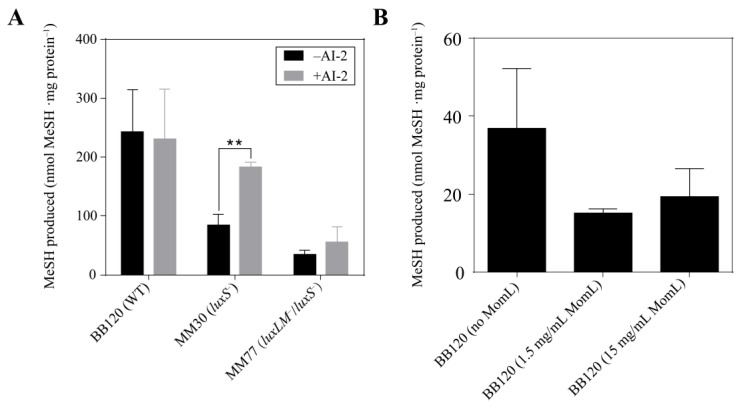
Production of MeSH by *Vibrio harveyi* cultured in MBM with autoinducers of different levels. (**A**) Production of MeSH by *V. harveyi* BB120, MM30, MM77 with/without AI-2 added. (**B**) Production of MeSH by *V. harveyi* BB120 with degrading enzyme MomL of different concentrations added. The data are shown as the mean ± standard deviation (SD). The differences between the experimental groups and the control groups were calculated by Student’s *t*-test. **, *p* < 0.01 in Student’s *t*-test.

To confirm the influence of QS on the MeSH production of *V. harveyi*, autoinducer AI-2 was supplemented to *V. harveyi* WT BB120, *luxS*^−^ mutant MM30 and *luxLM*^−^/*luxS*^−^ mutant MM77 (Figure 5A). Addition of AI-2 to the WT showed no effect on MeSH production. By contrast, MeSH produced by the AI-2 synthetase-encoding gene *luxS* mutant MM30 significantly increased MeSH mg total protein^−1^ from 87 to 185 nmol (*p* < 0.01), indicating that the addition of exogenous AI-2 offset the loss of *luxS* to some extent. Additionally, an insignificant increase in MeSH production was observed in the mutant MM77 (from 37 to 58 nmol MeSH mg total protein^−1^). This result implied that the reduced MeSH production caused by deficiency in both HAI-1 and AI-2 cannot simply be recovered by AI-2 only.

To further investigate the effect of the HAI-1 QS system on MeSH production, the quorum-quenching (QQ) enzyme MomL, which can degrade both short- and long-chain AHLs, was used to inhibit the HAI-1 QS system. The results showed that MomL at both 1.5 mg/mL and 15 mg/mL led to reduced MeSH production by ~50% (Figure 5B), although this was statistically insignificant. Higher concentrations of MomL did not lead to a stronger inhibition effect of MeSH production, possibly due to MomL at 1.5 mg/mL being sufficient to degrade all HAI-1 produced by *V. harveyi* BB120.

### 3.5. Growth Inhibition Caused by L-Met on V. harveyi BB120 and Its QS Mutants

**Figure 6:**
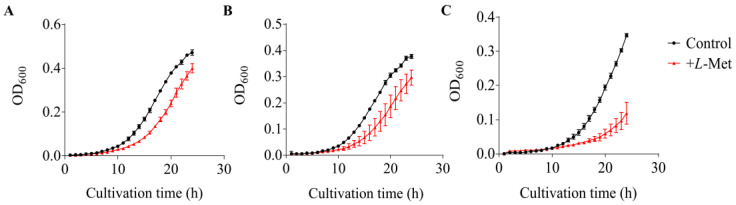
The growth curves of *Vibrio harveyi* cultured in MBM under different conditions. (**A**) Growth curves of *V. harveyi* BB120. (**B**) Growth curves of *V. harveyi* MM30. (**C**) Growth curves of *V. harveyi* MM77. The data are shown as the mean ± standard deviation (SD).

It has been reported that excess Met accumulated in the cell may be harmful for the organisms [66,67]. Therefore, converting Met to the volatile MeSH may help bacteria to regulate cellular Met levels. Here, we tested the growth inhibition effect of *L*-Met on *V. harveyi* WT BB120 and its QS mutants (MM30 and MM77) with varied MeSH production activity (Figure 6). Slight differences in the growth of BB120, MM30 and MM77 were observed, with the final OD_600_ after cultivation for 24 h reaching 0.472, 0.377 and 0.347, respectively. *L*-Met resulted in growth inhibition at 24 h on BB120, MM30 and MM77, and the final biomass reduced by ~15%, 20% and 66%, respectively. A more severe impairment of growth was observed in the *luxLM*^−^/*luxS*^−^ mutant MM77, which exhibited the weakest MeSH production activity among these three strains. These results indicated that the deficiency in the QS systems of *V. harveyi* may lead to reduced MeSH production and further reduced tolerance to a high Met level.

## 4. Discussion

MeSH and DMS are important volatile gases involved in the marine organic sulfur cycle and the regulation of climate [68,69,70,71]. Although widely distributed in diverse marine environments, whether these vibrios play roles as MeSH and DMS producers remains largely unknown. In this study, we examined the production of MeSH and DMS in the model vibrio strain *V. harveyi* BB120 and analyzed the distribution of potential genes related to *L*-Met and DMSP cycling in other vibrio genomes. On this basis, we further investigated the role of the QS cascade in regulating MeSH production of *V. harveyi* BB120 using a series of QS mutants, an autoinducer and a QQ enzyme. Our results indicated that MeSH production is widespread in vibrios and can be influenced by the complicated QS cascade in *V. harveyi*, especially the HAI-1 and AI-2 QS systems, which provides a novel insight into the production and regulation of this important organic sulfur compound.

### 4.1. Metabolism of Organic Sulfur in Vibrios

The microbial organic sulfur cycling involves active conversions between DMSP, DMS, MeSH and other sulfur compounds [72,73,74,75]. By screening the genomes of *V. harveyi* BB120 and other vibrios, we found that almost all vibrios are capable of MeSH production from *L*-Met through the *L*-methionine γ-lyase MegL. Although MegL widely exists in diverse bacteria [21,45,46,47,48,49], this is the first time this specific enzyme in vibrios has been focused on. Indeed, *V. harveyi* BB120 exhibited strong MeSH production when supplemented with *L*-Met. *L*-Met is an important amino acid involved in many cellular biochemical processes, information on the abundance and dynamics of dissolved Met in oceanic waters is scarce because of the methodological difficulties in its detection [76]. Therefore, the ecological importance of MeSH production from *L*-Met in these vibrios needs to be further investigated.

Microbial DMSP demethylation is another important source of MeSH [15]. However, homologs of known DMSP demethylase DmdA were absent in these vibrio genomes, and only a little MeSH was produced from DMSP in *V. harveyi* BB120, indicating that most vibrios were incapable of DMSP demethylation. Homologs of the DMSP lyases involved in DMSP cleavage, which produces DMS, were also seldom found in the *Vibrio* genomes (4/51). However, slight DMS production in *V. harveyi* BB120 indicated that there might be a novel DMSP lyase in BB120 which needs to be identified. Considering these results, vibrios may not be dominant DMSP consumers in the ocean, but some of them are possibly active in DMSP cleavage. Additionally, only a few homologs of genes involved in DMSP cycling were retrieved, except for DorA and MegL, indicating that DMSO reduction and MeSH production are the most prevalent organic sulfur metabolism processes in vibrios.

### 4.2. QS Systems Regulate MeSH Metabolism in Vibrio harveyi

*V. harveyi* BB120 was an established model strain for studying the complex QS systems in vibrios [77]. In this study, we found this strain was capable of MeSH production from *L*-Met, and this ability exhibited significant differences in varied QS mutants of *V. harveyi* BB120. To be specific, mutations in *luxLM* related to the HAI-1 pathway and *luxS*/*Q* related to the AI-2 pathway can cause a dramatic decrease in MeSH production; other genes located in the central QS regulation cascade, such as *luxO* and *hfq*, can also regulate MeSH release. These results provide the first evidence showing that the hierarchical QS systems in *V. harveyi* BB120 are comprehensively involved in the regulation of MeSH production. Indeed, when we supplied an AI-2 molecule to the *luxS* mutant, the MeSH production was partially recovered. Similarly, when we used a QQ enzyme to inhibit the HAI-1 QS pathway, the MeSH production reduced as expected. We proposed that the activation of the central QS cascade involving *luxO* is important for MeSH production in *V. harveyi* BB120. A deficiency in the HAI-1, AI-2 or CAI-1 QS pathway can also lead to reduced MeSH production, and inhibition of any of the two QS pathways mentioned above may intensify such reduction, as shown by the *luxLM*/*luxS*, *luxN*/*luxQ* and *luxN*/*cqsS* double mutants.

Although the QS systems of vibrios have been extensively investigated in numerous studies, most of them focused on the influence of QS on bioluminescence, biofilm formation, virulence factor production and secondary metabolite production [78,79,80,81]. We assumed that the involvement of QS in MeSH production is beneficial for vibrios, cooperatively controlling metabolic flux and relieving the stress caused by toxic metabolite accumulation, as proposed by Hawver et al. [82]. Indeed, we found that a high concentration of *L*-Met can inhibit the growth of *V. harveyi* BB120, and this inhibition effect was greater in QS mutants which were less capable of MeSH production. These results provide novel insights into the importance of QS and the MeSH production process.

## 5. Conclusions

Vibrios are extensively involved in converting *L*-Met to MeSH, and this process was regulated by the QS cascade in *V. harveyi* BB120. The HAI-1, AI-2 and CAI-1 QS pathways, as well as *luxO* and *hfq*, located in the center of this QS cascade, can affect MeSH production in *V. harveyi* BB120. Additionally, the QS regulation of MeSH production may be beneficial for vibrios when dealing with accumulated *L*-Met which may lead to growth inhibition.

## Data Availability

Data are contained within the article and Supplementary Materials.

## Supplementary Materials

The following are available online at https://www.mdpi.com/article/10.3390/microorganisms12010035/s1. Table S1. Strains for experiment and the description of their characteristics. Table S2. Standards used for the search of the potential functional proteins involved in MeSH and DMS metabolism by BLASTp. Table S3. The comparison results between MegL/DorAs in *Vibrio harveyi* BB120 and the functionally verified MegLs/DorAs. Table S4. Potential functional proteins involved in *L*-Met, DMSP, MeSH and DMS metabolism in typical *Vibrio* strains. Figure S1. Production of sulfur gas by *Vibrio harveyi* BB120 cultured in MA under different treatments. Figure S2. Maximum-likelihood phylogenetic tree of Dmd-type proteins (DmdB and AcuH) in *Vibrio*. Figure S3. Maximum-likelihood phylogenetic tree of Ddd-type proteins (DddD and DddP) in *Vibrio*. Figure S4. Maximum-likelihood phylogenetic tree of DdhA in *Vibrio*. Figure S5. Maximum-likelihood phylogenetic tree of DorAs in *Vibrio*.
