# Supplementary material for: Quorum Sensing Regulates the Production of Methanethiol in Vibrio harveyi
Source: Microorganisms. 2023 Dec 24;12(1):35. doi: 10.3390/microorganisms12010035 (PMC10819757; doi:10.3390/microorganisms12010035)
Supplement: Supplementary file 1 [file microorganisms-12-00035-s001.zip › microorganisms-2755458-supplementary.pdf]

Supplementary Material

Supplementary Tables

Table S1. Strains for experiment and the description of their characteristics.

Strains	Description of characteristics
BB120 (Bassler *et al.*, 1997)	*Vibrio harveyi* standard wild type strain
BB170 (Bassler *et al.*, 1993)	*luxN*::Tn5 (Kn^R) (HAI-1 sensor deactivated)
BB886 (Bassler *et al.*, 1994)	*luxQ*::Tn5 (Kn^R) (AI-2 sensor deactivated)
JAF375 (Freeman and Bassler, 1999)	*luxQ*::Tn5(Kn^R), *luxN*::Cm^R (HAI-1, AI-2 sensor deactivated)
JMH597 (Henke and Bassler, 2004a)	*luxN*::Tn5(Kn^R), *cqsS*::Cm^R (AI-2, CAI-1 sensor deactivated)
BB152 (Bassler *et al.*, 1994)	*luxLM*::Tn5 (Kn^R) (HAI-1 synthetase deactivated)
MM30 (Surette *et al.*, 1999)	*luxS*::Tn5(Kn^R) (AI-2 synthetase deactivated)
MM77 (Mok *et al.*, 2003)	*luxLM*::Tn5(Kn^R), *luxS*::Cm^R(HAI-1, AI-2synthetase deactivated)
JAF483 (Freeman and Bassler, 1999)	*luxO* D47A linked to Kn^R(LuxO point mutant)
BNL258 (Lenz *et al.*, 2004)	*hfq*::Tn5(Kn^R) lacZ (RNA chaperone *hfq* deactivated)

Table S2. Standards used for the search of the potential functional proteins involved in MeSH and DMS metabolism by BLASTp.

Protein	*e*-value cut-offs	Reference or detailed information
DsyB	$\leq e^{-67}$	Curson *et al.*, 2017
MmtN	$\leq e^{-50}$	Williams *et al.*, 2019
DmdA	$< e^{-85}$	Curson *et al.*, 2011b
DddP	$< e^{-86}$	Curson *et al.*, 2011b
DddL	$< e^{-52}$	Curson *et al.*, 2011b
DddD	0.0	Curson *et al.*, 2011b
DddQ	$< e^{-20}$	Curson *et al.*, 2011b
DddW	$< e^{-49}$	Curson *et al.*, 2011b
MegL	$< e^{-80}$	Identity > 40%, Coverage > 90%
DmdB	0.0	Identity > 49%, Coverage > 99%
DmdC	$< e^{-43}$	Identity > 33%, Coverage > 70%
DmdD/AcuH	$< e^{-108}$	Identity > 60%, Coverage > 98%
DddK	$< e^{-7}$	Identity > 39%, Coverage > 95%

(Continued)

Protein	*e*-value cut-offs	Reference or detailed information
DddX	$< e^{-75}$	Identity > 30%, Coverage > 70%
MddA	$< e^{-9}$	Identity > 36%, Coverage > 70%
DmoA	$< e^{-83}$	Identity > 36%, Coverage > 96%
DdhA	$< e^{-80}$	Identity > 36%
DorA	0.0	Identity > 43%, Coverage > 99%
DddY	$< e^{-5}$	-
DdhA	$< e^{-5}$	-
Tmm	$< e^{-5}$	-
MTO	$< e^{-5}$	-

Table S3. The comparison results between MegL/DorAs in *Vibrio harveyi* BB120 and the functionally verified MegLs/DorAs.

Query sequence	Subject sequence	Identity	*e*-value	Coverage
VH2527 (WP_005432739.1)	*Brevibacterium linens* (AAV54600.1)	44.845%	$4.26\times e^{-113}$	90.82%
	Clostridium tetani 157.15 (WP_129010005.1)	40.722%	$4.99\times e^{-93}$	96.98%
	Porphyromonas gingivalis LyG-1 (WP_211600051.1)	40.206%	$8.57\times e^{-87}$	96.74%
	Micromonospora echinospora	40.110%	$6.13\times e^{-82}$	99.23%
VH851 (WP_012127641.1)	DorA_*Rhodobacter sphaeroides* 2.4.1T (AAB94874.1)	45.000%	0.0	99.76%
	DorA_*Rhodobacter capsulatus* 37b4 (AAD13674.1)	44.567%	0.0	99.64%
VH4908 (WP_012129114.1)	DorA_*Rhodobacter sphaeroides* 2.4.1T (AAB94874.1)	40.438%	0.0	98.30%

Table S4. Potential functional proteins involved in *L*-Met, DMSP, MeSH and DMS metabolism in typical *Vibrio* strains.

Strains	Numbers of the potential functional proteins																					
	L-Met metabolism	DMSP synthesis		DMSP demethylation					DMSP cleavage								MeSH→DMS	DMS→MeSH	DMS→DMSO		DMSO→DMS	MeSH→HCHO
	MegL	DsyB	MmtN	DmdA	DmdB	DmdC	DmdD	AcuH	DddD	DddL	DddP	DddQ	DddW	DddY	DddK	DddX	MddA	DmoA	DdhA	Tmm	DorA	MTO
Vibrio aerogenes LMG 19650	1	0	0	0	0	0	0	0	0	0	0	0	0	0	0	0	0	0	0	0	0	0
Vibrio antiquarius EX25	1	0	0	0	0	0	0	0	0	0	0	0	0	0	0	0	0	0	0	0	1	0
Vibrio aquimaris THAF100	1	0	0	0	0	0	0	0	0	0	0	0	0	0	0	0	0	0	0	0	1	0
Vibrio artabrorum CECT 7226	1	0	0	0	0	0	0	0	0	0	0	0	0	0	0	0	0	0	0	0	1	0
Vibrio atlanticus CECT 7223	1	0	0	0	0	0	0	0	0	0	0	0	0	0	0	0	0	0	0	0	1	0
***Vibrio bathopelagicus* Sal10**	1	0	0	0	0	0	0	0	0	0	1	0	0	0	0	0	0	0	0	0	1	0
Vibrio campbellii BoB-53	1	0	0	0	0	0	0	0	0	0	0	0	0	0	0	0	0	0	0	0	1	0
Vibrio chagasii LMG 21353	1	0	0	0	0	0	0	0	0	0	0	0	0	0	0	0	0	0	0	0	1	0
Vibrio cholerae RFB16	1	0	0	0	0	0	0	0	0	0	0	0	0	0	0	0	0	0	0	0	1	0
Vibrio coralliirubri DSM 27495	1	0	0	0	0	0	0	0	0	0	0	0	0	0	0	0	0	0	0	0	1	0
Vibrio cortegadensis CECT 7227	1	0	0	0	0	0	0	0	0	0	0	0	0	0	0	0	0	0	0	0	1	0
Vibrio diabolicus HS-60-3	1	0	0	0	0	0	0	0	0	0	0	0	0	0	0	0	0	0	0	0	1	0
Vibrio echinoideorum DSM 107264	1	0	0	0	0	0	0	0	0	0	0	0	0	0	0	0	0	0	0	0	1	0
Vibrio fluvialis ATCC 33809	1	0	0	0	0	0	0	0	0	0	0	0	0	0	0	0	0	0	0	0	1	0
Vibrio fortis LMG 21557	1	0	0	0	0	0	0	0	0	0	0	0	0	0	0	0	0	0	0	0	1	0
***Vibrio furnissii* FDAARGOS_777**	1	0	0	0	1	0	0	0	0	0	0	0	0	0	0	0	0	0	0	0	1	0
Vibrio galatheae S2757	1	0	0	0	0	0	0	0	0	0	0	0	0	0	0	0	0	0	0	0	1	0
Vibrio gallaecicus CECT 7244	1	0	0	0	0	0	0	0	0	0	0	0	0	0	0	0	0	0	0	0	1	0
Vibrio gangliei DSM 104291	1	0	0	0	0	0	0	0	0	0	0	0	0	0	0	0	0	0	0	0	0	0
***Vibrio gigantis* ACE001**	1	0	0	0	0	0	0	0	0	0	1	0	0	0	0	0	0	0	0	0	1	0
Vibrio ishigakensis C1	1	0	0	0	0	0	0	0	0	0	0	0	0	0	0	0	0	0	0	0	1	0
Vibrio japonicus JCM 31412	1	0	0	0	0	0	0	0	0	0	0	0	0	0	0	0	0	0	0	0	1	0
Vibrio kanaloae R17	1	0	0	0	0	0	0	0	0	0	0	0	0	0	0	0	0	0	0	0	1	0
Vibrio mangrovi CECT 7927	1	0	0	0	0	0	0	0	0	0	0	0	0	0	0	0	0	0	0	0	0	0
Vibrio marisflavi CECT 7928	1	0	0	0	0	0	0	0	0	0	0	0	0	0	0	0	0	0	0	0	0	0
***Vibrio mediterranei* Vic-OC-097**	1	0	0	0	1	0	0	0	0	0	1	0	0	0	0	0	0	0	0	0	1	0
***Vibrio natriegens* NBRC 15636**	1	0	0	0	1	0	0	0	0	0	0	0	0	0	0	0	0	0	0	0	1	0
Vibrio neonatus JCM 21521	1	0	0	0	0	0	0	0	0	0	0	0	0	0	0	0	0	0	0	0	0	0
Vibrio nereis NBRC 15637	1	0	0	0	0	0	0	0	0	0	0	0	0	0	0	0	0	0	0	0	1	0
***Vibrio ostreae* OG9-811**	1	0	0	0	0	0	0	0	1	0	0	0	0	0	0	0	0	0	0	0	0	0
Vibrio owensii XSBZ03	1	0	0	0	0	0	0	0	0	0	0	0	0	0	0	0	0	0	0	0	1	0
Vibrio palustris CECT 9027	1	0	0	0	0	0	0	0	0	0	0	0	0	0	0	0	0	0	0	0	0	0
Vibrio parahaemolyticus RIMD 2210633	1	0	0	0	0	0	0	0	0	0	0	0	0	0	0	0	0	0	0	0	1	0
Vibrio pelagius ATCC 25916	1	0	0	0	0	0	0	0	0	0	0	0	0	0	0	0	0	0	0	0	1	0
***Vibrio penaeicida* IFO 15640T**	1	0	0	0	0	0	0	1	0	0	0	0	0	0	0	0	0	0	0	0	1	0
Vibrio pomeroyi LMG 20537	1	0	0	0	0	0	0	0	0	0	0	0	0	0	0	0	0	0	0	0	1	0
***Vibrio ponticus* DSM 16217**	1	0	0	0	1	0	0	0	0	0	0	0	0	0	0	0	0	0	0	0	1	0
Vibrio porteresiae DSM 19223	1	0	0	0	0	0	0	0	0	0	0	0	0	0	0	0	0	0	0	0	0	0
Vibrio qinghaiensis Q67	1	0	0	0	0	0	0	0	0	0	0	0	0	0	0	0	0	0	0	0	1	0
Vibrio rarus LMG 23674	1	0	0	0	0	0	0	0	0	0	0	0	0	0	0	0	0	0	0	0	1	0
Vibrio rhizosphaerae LMG 23790	1	0	0	0	0	0	0	0	0	0	0	0	0	0	0	0	0	0	0	0	0	0
Vibrio rotiferianus B64D1	1	0	0	0	0	0	0	0	0	0	0	0	0	0	0	0	0	0	0	0	1	0
Vibrio rumoiensis FERM P-14531	1	0	0	0	0	0	0	0	0	0	0	0	0	0	0	0	0	0	0	0	0	0
Vibrio sagamiensis NBRC 104589	1	0	0	0	0	0	0	0	0	0	0	0	0	0	0	0	0	0	0	0	1	0
Vibrio splendidus 2_C04b	1	0	0	0	0	0	0	0	0	0	0	0	0	0	0	0	0	0	0	0	1	0
Vibrio tasmaniensis LMG 20012	1	0	0	0	0	0	0	0	0	0	0	0	0	0	0	0	0	0	0	0	1	0
Vibrio toranzoniae CECT 7225	1	0	0	0	0	0	0	0	0	0	0	0	0	0	0	0	0	0	0	0	1	0
***Vibrio tritonius* AM2**	1	0	0	0	0	0	0	0	0	0	0	0	0	0	0	0	0	0	1	0	0	0
Vibrio tubiashii ATCC 19109	1	0	0	0	0	0	0	0	0	0	0	0	0	0	0	0	0	0	0	0	1	0
***Vibrio zhugei* HBUAS61001**	1	0	0	0	0	0	0	0	0	0	0	0	0	0	0	0	0	0	1	0	0	0
Vibrio ziniensis ZWAL4003	1	0	0	0	0	0	0	0	0	0	0	0	0	0	0	0	0	0	0	0	0	0

Supplementary Figures

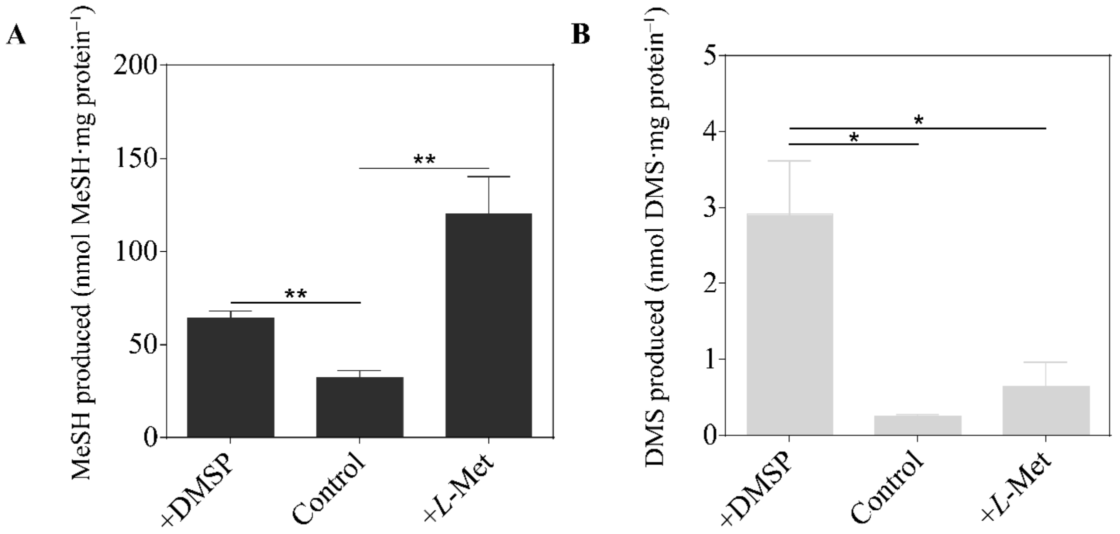

Figure S1. Production of sulfur gas by *Vibrio harveyi* BB120 cultured in MA under different treatments. A, Production of MeSH by *V. harveyi* BB120. B, Production of DMS by *V. harveyi* BB120. The data are shown as the mean ± standard deviation (SD). The differences between the experimental groups and the control groups were calculated by Student's *t*-test. *, $p < 0.05$ in Student's *t*-test. **, $p < 0.01$ in Student's *t*-test.

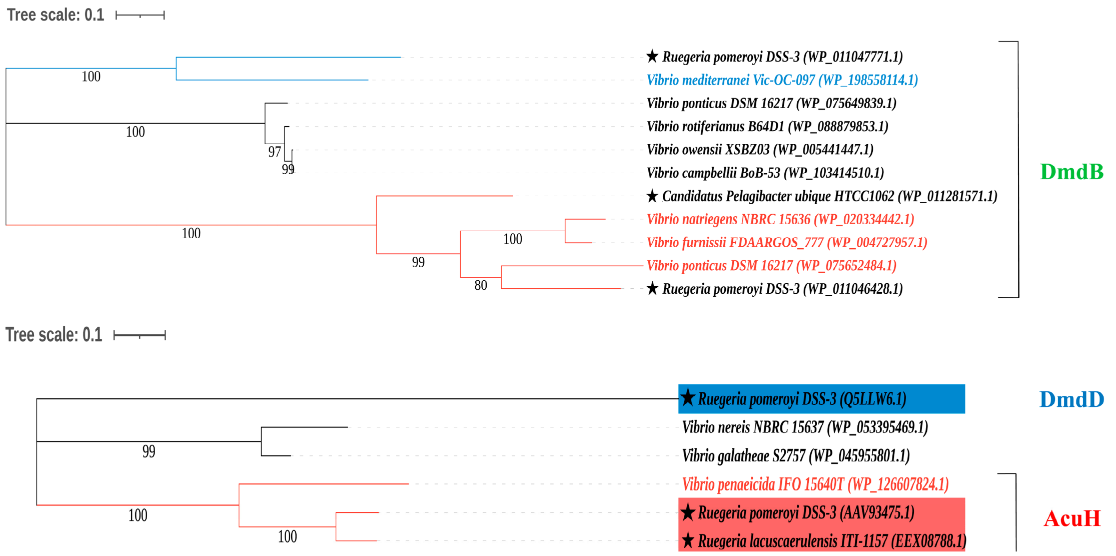

Figure S2. Maximum-likelihood phylogenetic tree of Dmd-type proteins (DmdB and AcuH) in *Vibrio*. Ratified DmdB and AcuH/DmdD alongside the proteins of the representative vibrios available from NCBI that sharing higher identity with them were used for phylogenetic tree construction. The ratified proteins were marked with a black star and those in red/blue were potential functional proteins. Bootstrap support for nodes was marked.

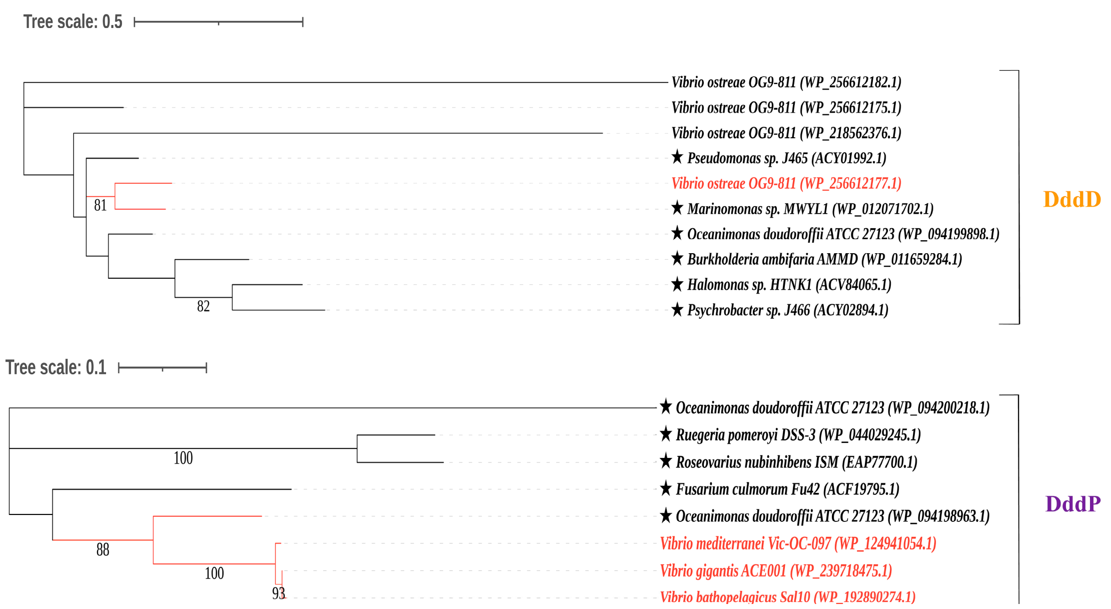

Figure S3. Maximum-likelihood phylogenetic tree of Ddd-type proteins (DddD and DddP) in *Vibrio*. Ratified DddD and DddP alongside the proteins of the representative vibrios available from NCBI that sharing higher identity with them were used for phylogenetic tree construction. Proteins experimentally confirmed to produce DMS were marked with a black star and those in red were potential DMSP lyases. Bootstrap support for nodes was marked.

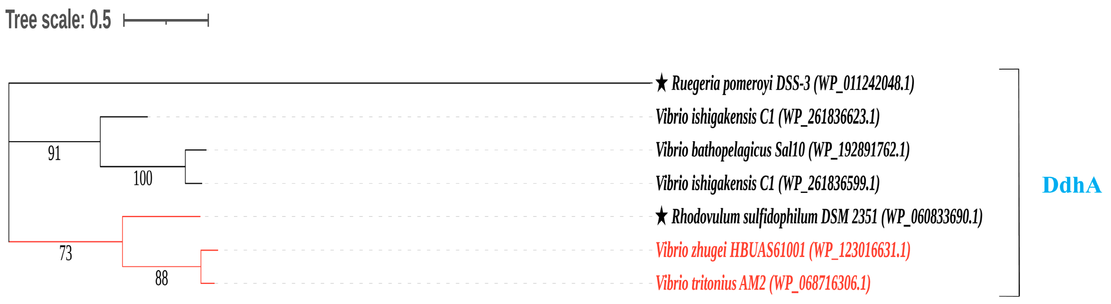

Figure S4. Maximum-likelihood phylogenetic tree of DdhA in *Vibrio*. Ratified DdhA alongside the proteins of the representative vibrios available from NCBI that sharing higher identity with them were used for phylogenetic tree construction. The ratified proteins experimentally confirmed to transform DMS to DMSO were marked with a black star and those in red were potential DdhAs. Bootstrap support for nodes was marked.

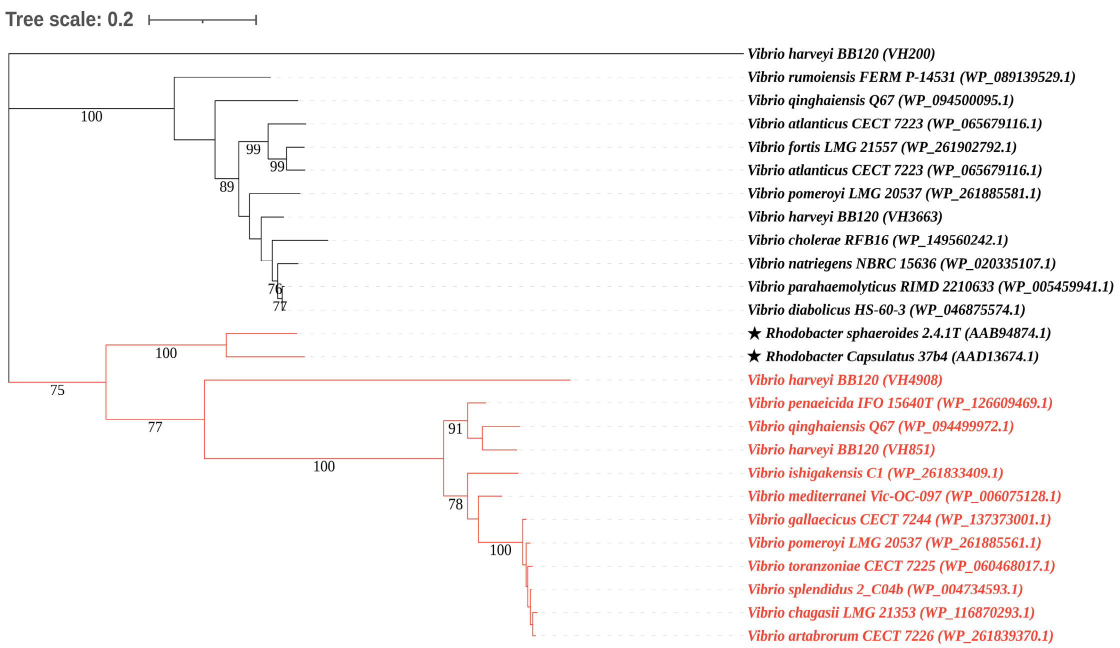

Figure S5. Maximum-likelihood phylogenetic tree of DorAs in *Vibrio*. Ratified DorAs alongside the proteins of the representative vibrios available from NCBI that sharing higher identity (Top 10) with them were used for phylogenetic tree construction. Proteins experimentally confirmed to transform DMSO to DMS were marked with a black star and those in red were potential DorAs. Bootstrap support for nodes was marked.

Reference

Bassler, B. L., Wright, M., Showalter, R. E., and Silverman, M. R. (1993). Intercellular signalling in *Vibrio harveyi*: sequence and function of genes regulating expression of luminescence. *Mol. Microbiol.* doi:10.1111/j.1365-2958.1993.tb01737.x.

Bassler, B. L., Wright, M., and Silverman, M. R. (1994). Multiple signalling systems controlling expression of luminescence in *Vibrio harveyi*: sequence and function of genes encoding a second sensory pathway. *Mol. Microbiol.* doi:10.1111/j.1365-2958.1994.tb00422.x.

Bassler, B. L., Greenberg, E. P., and Stevens, A. M. (1997). Cross-species induction of luminescence in the quorum sensing bacterium *Vibrio harveyi*. *J. Bacteriol.* doi:10.1128/jb.179.12.4043-4045.1997.

Curson, A. R. J., Liu, J., Bermejo Martínez, A., Green, R. T., Chan, Y., Carrión, O., et al. (2017). Dimethylsulfoniopropionate biosynthesis in marine bacteria and identification of the key gene in this process. *Nat. Microbiol.* doi:10.1038/nmicrobiol.2017.9.

Curson, A. R. J., Todd, J. D., Sullivan, M. J., and Johnston, A. W. B. (2011b). Catabolism of dimethylsulphoniopropionate: microorganisms, enzymes and genes. *Nat. Rev. Microbiol.* doi:10.1038/nrmicro2653.

Freeman, J. A., and Bassler, B. L. (1999). A genetic analysis of the function of LuxO, a two-component response regulator involved in quorum sensing in *Vibrio harveyi*. *Mol. Microbiol.* doi:10.1046/j.1365-2958.1999.01208.x.

Henke, J. M., and Bassler, B. L. (2004a). Three parallel quorum-sensing systems regulate gene expression in *Vibrio harveyi*. *J. Bacteriol.* doi:10.1128/jb.186.20.6902-6914.2004.

Lenz, D. H., Mok, K. C., Lilley, B. N., Kulkarni, R. V., Wingreen, N. S., and Bassler, B. L. (2004). The small RNA chaperone Hfq and multiple small RNAs control quorum sensing in *Vibrio harveyi* and *Vibrio cholerae*. *Cell.* doi:10.1016/j.cell.2004.06.009.

Mok, K. C., Wingreen, N. S., and Bassler, B. L. (2003). *Vibrio harveyi* quorum sensing: a coincidence detector for two autoinducers controls gene expression. *EMBO J.* doi:10.1093/emboj/cdg085.

Surette, M. G., Miller, M. B., and Bassler, B. L. Quorum sensing in *Escherichia coli*, *Salmonella typhimurium*, and *Vibrio harveyi*: A new family of genes responsible for autoinducer production. *Proc. Natl. Acad. Sci.* doi:10.1073/pnas.96.4.1639.

Williams, B. T., Cowles, K., Bermejo Martínez, A., Curson, A. R. J., Zheng, Y., Liu, J., et al. (2019). Bacteria are important dimethylsulfoniopropionate producers in coastal sediments. *Nat. Microbiol.* doi:10.1038/s41564-019-0527-1.
